# Model-Based Reinforcement Learning with Automated Planning for Network Management

**DOI:** 10.3390/s22166301

**Published:** 2022-08-22

**Authors:** Armando Ordonez, Oscar Mauricio Caicedo, William Villota, Angela Rodriguez-Vivas, Nelson L. S. da Fonseca

**Affiliations:** 1 Universidad ICESI, Cali 760031, Colombia; 2 Departamento de Telematica, Universidad del Cauca, Popayan 190002, Colombia; 3 Institute of Computing, University of Campinas, Campinas 13083-852, Brazil

**Keywords:** automated planning, model based, reinforcement learning, network management

## Abstract

Reinforcement Learning (RL) comes with the promise of automating network management. However, due to its trial-and-error learning approach, model-based RL (MBRL) is not applicable in some network management scenarios. This paper explores the potential of using Automated Planning (AP) to achieve this MBRL in the functional areas of network management. In addition, a comparison of several integration strategies of AP and RL is depicted. We also describe an architecture that realizes a cognitive management control loop by combining AP and RL. Our experiments evaluate on a simulated environment evidence that the combination proposed improves model-free RL but demonstrates lower performance than Deep RL regarding the reward and convergence time metrics. Nonetheless, AP-based MBRL is useful when the prediction model needs to be understood and when the high computational complexity of Deep RL can not be used.

## 1. Introduction

Reinforcement Learning (RL) has been used recently in network management for automating diverse tasks such as traffic monitoring and routing [1,2,3,4]. However, most of theses applications require a huge amount of samples from the environment. This trial and error process implies that some non-optimal or wrong actions can be performed during the learning process, which is unacceptable in real scenarios with strong Service-Level Agreements (SLA) [5].

Deep Reinforcement Learning (DRL) aims at addressing some of these shortcomings while offering unique advantages in terms of accuracy. However, DRL has its own drawbacks, first the success of DRL depends on the the availability of data [6]. Second, DRL is vulnerable to adversarial examples [7]. Third, the tuning of the hyper-parameters may be very complex and typically requires a higher convergence time than RL [8]. Finally, DRL results in black boxes that are hard to interpret [9]. Therefore, in some real network management scenarios, alternative solutions are preferred even at the expense of accuracy.

Existing AI-based approaches for network management automation focus on simple tasks (i.e., selecting a route). Thus, many complex tasks in network management are still manual. However, to achieve self-driving networks, it is required to automatically perform a broad set of tasks. For example, in the Radio Access Network, some tasks such as the adjustment of tilt, frequency, or radiation pattern need to be carried out automatically to achieve adaptability in cellular networks [10]. In network slicing, some of these tasks may include on-demand provisioning, migration, or scaling up/down of virtual network functions (VNFs). These tasks must be performed in order and achieve a predefined goal (maintain QoE, optimize costs).

In addition, networks architectures are evolving continually; therefore, it is necessary to perform diverse management tasks for every architecture and technology. This process requires a technique capable of finding the best sequence of tasks (plans) necessary to achieve a desired solution (i.e., maximizing a long-term reward). In addition, in these diverse architectures, sometimes there are not enough data available for training neural networks.

An approach for these types of Markov Decision Process (MDP) optimization problems [11] is automated planning (AP). AP builds plans to achieve a particular goal status. To come up with these plans, AP uses a model of the environment called a domain. AP offers various advantages such as expressiveness and simplicity to represent the environment [12].

Due to their similarities, AP and RL has been combined to model-based reinforcement learning [13]. Unlike model-free RL methods that learn just by interacting with the environment, MBRL methods model the environment, which reduces the number of interactions needed to learn [13,14].

MBRL approaches have evidenced similar performance to model-free algorithms in diverse domains but require significantly fewer interactions with the environment [15,16,17]. This paper explores the use of this concept in network management. Notably, we analyze how AP models (domains) can be used to represent the behavior and status on the network, and RL can exploit such representation to reduce the number of interactions with the environment [18]. In addition, we propose an architecture that realizes a cognitive management control loop by combining AP and RL. We evaluated our approach in a prototype that automates the network slicing admission control. Most of the existing approaches focus on using RL for punctual task such as network traffic detection [19] and load balancing [20]. However, to the best of our knowledge, a proposal describing how AP an RL can be integrated in practice for network management has not been presented.

Evaluation results in simulated environment evidence that the combination proposed improves RL but demonstrates lower performance than DRL regarding the reward and convergence time metrics. Consequently, this paper encourages the use of AP-based RL in scenarios where the complexity of neural networks cannot be faced.

The remainder of this paper is organized as follows. Section 3 presents a brief background about AP and RL in network management. Section 4 shows how some functional areas of network management can be automated using MBRL. Section 5 presents an architecture for cognitive management based on AP and RL for MBRL. Section 6 introduces a case study that shows how our approach can be used jointly for network slicing admission control. Section 7 concludes and explores the future work.

## 2. Methodology

After introducing some relevant concepts, we explore the state-of-the-art and propose diverse combination strategies for the use of MBRL in network management.

To validate the feasibility of our approach, we propose an architecture for cognitive management based on AP and RL.

Finally, based on this architecture, we implement and evaluate a case study for automating the admission control for network slicing using AP and RL.

We built a discrete event simulator for network slicing admission control. To do so, we deployed in such a simulator a 16-node network topology generated by using the Barabasi–Alberth algorithm [21]. This network was composed of 4 core nodes and 12 edge nodes, with capacities of 300 and 100 processing units, respectively. All substrate links had a capacity of 100 bandwidth units. We evaluated 80 episodes and 33 repetitions. The average Reward was analyzed.

## 3. Background: RL and AP in Network Management

### 3.1. Reinforcement Learning in Network Management

RL agents learn by interacting with the environment through a set of episodes. In each episode, an agent performs *actions* that change the environment state and then receives rewards [22]. The objective is to determine the optimal *action* in each *state* that allows optimizing a *goal* [23]. The goal can be to optimize the resource usage in a data center.

In network management, the environment can be, for example, a 5G/6G network. This environment (network) can be modeled as a Markov Decision Process [11] formed by states (e.g., one state can be, for example, a particular location of Virtual functions in substrate nodes), actions (e.g., to route packages or to up- or downscale a virtual network function), and rewards.

Model-free RL learns directly from experience by performing actions in the environment and collecting their respective rewards. These interactions are represented as episodes, and the experience from each episode is used for training a Policy or a Q-value function that can be used later in other episodes [24]. Model-free RL is hard to scale, and its use is constrained to low-dimensional problems. This is due to the high memory requirements and high computational cost of MFRL [25].

Deep Reinforcement learning (DRL) solves some of the issues mentioned above by combining RL and Deep learning (DL) [26,27]. In particular, DRL takes the structure and processes from RL, keeping a reinforcement behavior in the training phase, and neural networks (with a high number of layers) from DL to estimate the values for each state–action pair. DRL usually makes better decisions than RL and allows analyzing domains with a high number of states and actions [28].

Despite the advantages provided by DRL, this approach has many drawbacks in network management scenarios where there are not large datasets for training neural networks or where black-box approaches are not acceptable for decision making.

### 3.2. Automated Planning

AP aims at automatically creating plans (set of possible actions) to pass from an initial state (real-world situation) to a goal state (target status) [29].

For example, in a large-scale network handled by various Software-Defined Network (SDN) controllers, an unexpected service disruption occurs due to the root controller outage. This outage is the *initial state*, and the *goal state* is to meet a previously signed SLA. An example of *action* can be to replace the links of a data center branch with their backups or restart a controller while a mirror one operates in its representation, seeking to hold up the network availability within the SLA-allowed threshold.

The *initial state* can be obtained by monitoring the current network status, and the *goal state* can be specified by the network administrator or obtained from a network policy or a network intent [30]. The *initial state* and *goal state* are contained in a *planning problem*, and the set of feasible *actions* (describing real-world activities) that can be used to go from the *initial state* to the *goal state* are contained in a *planning domain* [29]. The *planning domain* is defined by the Network Administrator [31].

There exist different AP methods, and each one of them is suitable for a particular domain. For example, Markov decision-based planning (MDP) [32] uses trees instead of sequences for representing state transitions. Thus, MDP is the best option for non-deterministic scenarios (i.e., one action may produce different effects on the same state), such as in wireless networks where the topology may change dynamically. Temporal planning [33] offers a reliable alternative when multiple actions can be carried out simultaneously, as when diverse configurations need to be provisioned autonomously in an SDN handled by a distributed control plane.

Hierarchical Task Networks (HTN) [34] are useful when tasks are representable hierarchically, as in the restoring of a protocols stack during a fault-recovery process.

HTN has three types of tasks [35]. *Goal tasks* are properties that we would like to make true (e.g., achieving an specific value of latency or delay). *Primitive tasks* can be directly achieved by executing the corresponding action (e.g., turning a switch on). *Compound tasks* denote desired changes that involve several *goal tasks* and *primitive tasks* (e.g., deploying a slice) and require many other tasks to be performed (create the virtual machines, provision the nodes, etc.). A *compound task* allows us to represent a “desired change” that cannot be represented as a single *goal task* or *primitive task* [35].

HTN includes an initial state description, a *task network* as an objective to be achieved, and domain knowledge consisting of networks of *primitive* and *compound tasks*. A *task network* represents a hierarchy of tasks, each of which can be executed if the task is primitive or decomposed into refined subtasks. The planning process starts by decomposing the initial task network and continues until all *compound tasks* are decomposed. The solution is a plan which equates to a set of *primitive tasks* applicable to the initial world state [36].

There exist some conditions to apply AP to a particular application domains. First, the domain knowledge must be well structured to express *domain goals* and activities as *actions* and *plans*. Second, detailed domain information must exist for describing how to find a solution (achieve a goal) using a set of *actions* (plan). Network management meets these conditions because the network management functional areas are well-known and expressible by primitive tasks forming management plans [37].

AP shares the same philosophy of *Declarative models* from Devops technologies such as CloudFormation, Terraform, and Kubernetes [38].

Unlike imperative models where tasks are specified in a procedural manner, declarative models express the desired state into which an application needs to be transferred [39].

AP is capable of creating plans representing network management tasks, thus going further than the declarative models. *AP plans* can be optimized automatically according to a particular metric such as latency or CPU load (also known as a constraint). An example of this constraint in planning language is depicted below:

(:goal (and (at VNF1 middle-box9) (preference (off server2)))) (:metric minimize (* 10 (latency))).

In the previous snippet, predicates (properties of objects that can be true or false) represent real-world goals. In the example, the first predicate specifies that VNF1 must be installed on middle–box9. The second predicate illustrates a preference that indicates that the server2 must be turned off. As many plans can be created to reach the goal state, the third predicate specifies that the plan should be chosen based on the minimum latency.

### 3.3. Model-Based Reinforcement Learning

MBRL aims at reducing the interactions with the environment during the training. In MBRL, this reduction is achieved by using a model of the environment to simulate part of the episodes [17]. It is noteworthy that despite the fact that MBRL methods have recently evidenced similar performance as model-free RL with significantly fewer interactions with the environment [15,17], they have been used little in the network management domain. However, Effective MBRL may be cumbersome because the ease of data generation must be weighed against the bias of model-generated data [40].

## 4. Integrating Automated Planning and Reinforcement Learning for Network Management

Due to the fact that RL and AP share many similarities, several authors have proposed diverse ways to integrate RL and AP aiming at improve their individual performance in a MBRL approach [18,41,42]. This section describes diverse combination strategies for the use of MBRL in network management.

**Figure 1:**
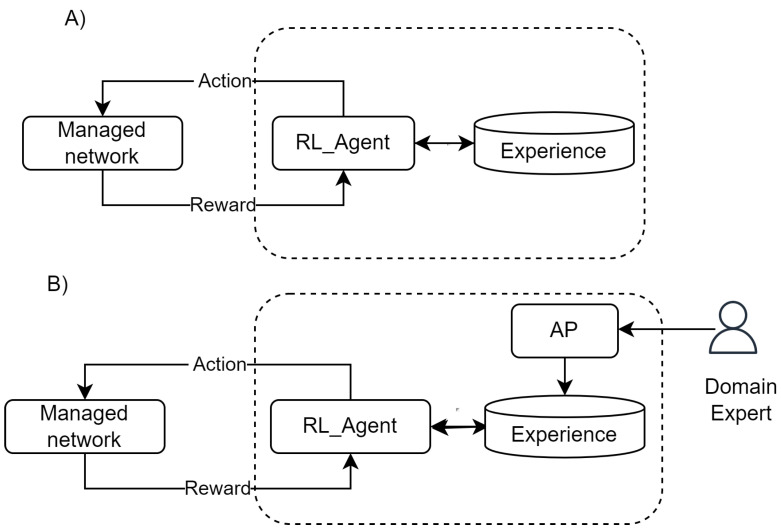
Integrating AP and RL.

Generally Speaking, the main idea is to reduce the interactions required to learn. Figure 1A depicts the basic RL functioning, and Figure 1B includes a new agent that feeds the experience with the results of simulations.

### 4.1. Combination Strategies

The strategy ***First Plan then Learn (1P2L)*** aims at achieving more complex tasks with lower learning time. To reduce the computational effort required to explore a broad set of actions and states, AP generates a plan formed by a set of actions with high-level of abstraction (macro-operators, such as *improve slice performance of virtual functions* or *optimize controller CPU consumption*). These macro-operators become the goal of RL that in turn deals with fewer (and with lower level of abstraction) actions. For example, in Radio Access Networks (RAN), a set of macro-operators may include *to route the traffic* or *calibrate the antenna azimuth*. In the latter case, the antenna calibration becomes the *goal* of the RL agent, and some examples of the granular actions may be to move left and move right.

The strategy ***First Learn then Plan (1L2P)*** is suitable for scenarios with high number of actions. It starts by applying RL; however, instead of analyzing all the possible actions, these are grouped in high-level actions (macro-operators); thus, the number of actions to explore is reduced. These macro-operators become the *goal state* of AP, which computes a plan with lower-abstraction actions, such as *install an specific software in a virtual machine* or *set particular configuration parameters*. *1L2P* can support more complex tasks in lower time. This strategy of combination can be used in situations where continuous sensing is difficult, unreliable, or undesirable. For example, when SLAs are very rigorous or where the channel bandwidth or processing capabilities are scarce such as in IoT-based scenarios.

In the strategy ***Interchange Learning and Planning (IXLP)***, a model of the world is created, and the value of each action-state of RL is updated by simulating AP transitions. This model can be created from experience in other domains and implemented in a table including all the possible states and the value of each action in each state. AP operates concurrently with an RL algorithm that updates the same value in the table (representing the model) by actual interactions with the environment. This coordinated operation helps RL to converge faster since the AP-based simulation of the interactions with the model is carried out much faster than the actual interactions. Similar to the previous strategies, this combination aims at reducing the learning time. *IXLP* can also be used in situations where continuous sensing is difficult, unreliable, or undesirable, such as dynamic SDN-based multipath routing, where continuous changes in the table flows of OpenFlow-enabled switches can lead to network instability.

### 4.2. MBRL in the Network Management of Functional Areas

Recent works have explored the individual use of RL, DRL, and AP for automation tasks in network management. However, these works face several issues, such as the computational effort required and the need for an efficient feedback from the environment.

In this section, we describe some examples of how to use MBRL in functional network management areas: Faults, Configuration, Accounting, Performance, and Security (FCAPS).

#### Faults Management

There are many faults-management solutions based on RL. For instance, RL-based algorithms have been used to manage faults in cellular networks [43,44]. Another solution for fault management is available in [45], which describes how an outdoor cellular network must address various faults, such as changes in the antenna azimuth due to wind or failures in the neighboring cells. In such a scenario, the goal is to maintain the Signal to Interference and Noise Ratio (SINR) within predefined values. The actions are, among others, to change the antenna azimuth and modify the transmission power. The reward is a function of the throughput and SINR. This solution can only address few actions mainly because the RL computational time grows regarding the number of the states and actions [10,46]. Furthermore, RL requires much interaction with the environment to learn the best actions.

The *IXPL* strategy is helpful to address the issues of the solution previously described. In particular, AP enables computing a cellular environment model and initializes the state–action pairs (table or function) of RL. Thus, the RL agent must deal with a smaller search space; RL’s complexity and learning time decrease as less exploration is required.

### 4.3. Configuration Management

An example of using AP for performing configuration management tasks is available in [37], where: (i) the initial state is scalability issues due to SDN controller limitations; (ii) the goal state is to deploy a new SDN controller without causing network service disruption; (iii) the planning domain comprises a set of tasks (e.g., turn on/off the controller and assigning the OpenFlow switches to the controller) that enable promptly configure the SDN-based network; and (iv) HTN is used to build up plans that will allow moving from the initial state to the goal state using the planning domain.

The *1P2L* strategy is helpful to enhance the HTN-based solution previously described. In particular, RL helps to select the optimal HTN plan that allows accomplishing the goal state (i.e., migrate a SDN controller without network service disruption). For the RL agent, the set of candidate plans can correspond to the space of actions. The space of states can be the network status during the deployment of the new controller. The reward can be a function of the migration and service disruption times.

### 4.4. Accounting

To the best of our knowledge, in the accounting management functional area, no work has been conducted with AP or RL and even less by combining them. Future investigations can combine AP and RL to, for instance, optimize billing plans in highly dynamic environments such as 5G network slicing.

Let us consider that a basic billing solution is AP-based. The initial state comprises information about the resources consumption in diverse network slices and the cost of each resource per slice and tenant. The goal state is a calculated billing. The planning domain includes as tasks the computations needed for pricing. Using the *1P2L* approach, the RL agent can receive as input basic billing plans and learn to optimize them by considering the actual consumption and tariffs.

### 4.5. Performance

There are many performance management solutions based on RL. Some admission control algorithms use RL to improve network utilization and maximize operators’ profit [47,48]. In addition, diverse RL-based routing solutions [49,50] employ RL to meet QoS. Particularly, the solution available in [4] uses a Q-learning agent to make optimal routing decisions in SDN, where (i) all switches on the data plane comprise the space of states; (ii) the space of actions of each state corresponds to its neighboring switches; (iii) the reward is a function involving link-state information; and (iv) the Q-table contains all candidate paths with their corresponding reward for all network nodes (pair state-actions). This solution’s learning and execution times increase with the Q-table size (depending on the number of switches in the data plane), limiting its use in large networks.

The *1P2L* strategy is helpful to decrease the times mentioned above. Initially, AP can optimize the Q-table size. The initial state corresponds to the network status and candidate paths. The goal state is to resize the set of state–action pairs leading to a decrease in the Q-table size. The planning domain includes the computations needed to rank all candidate paths and the most congested ones for each pair of nodes (switches) in the network. By using this reduced table, the agent of Q-learning could make faster and better decisions.

### 4.6. Security

There are several security management solutions based on RL [51,52] or AP [53] intended to identify attacks, such as denial of service (DoS), man-in-the-middle attacks, and malware on the network. In particular, the solution in [54] uses AP to automate penetration testing, which in turn allows for identifying potentially exploitable security weaknesses. Automated penetration testing (i.e., automated attack-finding) is carried out on a network model analyzing changes in the network topology, system updates, and configuration changes. AP allows determining optimal combinations that minimize the maximal attacker success.

The *1P2L* strategy is helpful to enhance the solution previously described. Mainly, RL helps to select the optimal plan that allows for identifying attacks efficiently (goal state). For the RL agent, the set of candidate plans to address an attack can correspond to the space of actions. The space of states can be the network status during the security attack. The reward can be a function of the outage time caused by the attack.

## 5. An Architecture for Cognitive Management Based on AP and RL

**Figure 2:**
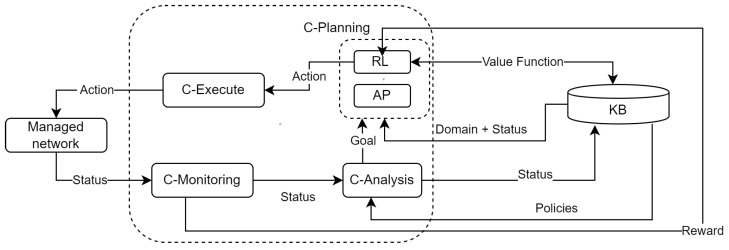
Automated Planning and Reinforcement Learning in the C-MAPE model.

Figure 2 shows the architecture proposed to combine AP and RL in the Cognitive MAPE (C- MAPE) introduced in [55]. We consider this proposal as an essential step in the path to achieve Zero-Touch Management [56] that aims at automating configuration, monitoring, and control tasks in large-scale networks to obtain a whole end-to-end architecture framework designed for closed-loop automation without requiring human intervention.

All the MAPE functions share the *Knowledge base* (KB). This KB stores all the information of the network as well as the information needed by RL and AP agents to perform their tasks, including: (i) the network state at any time (e.g., configurations, load of switches, and routers, available bandwidth, and delay of links) and its corresponding models (i.e., initial state for AP, and space of states for RL); (ii) the set of possible actions to carry out (i.e., problem domain for AP and space of actions for RL); (iii) the set of policies that govern the network management (i.e., target state for AP and rewards for RL agents); and (iv) the AP plans and RL models built, which can be optimized by the combination strategies discussed in Section 4.1.

*C-Monitoring* collects and measures information about the network status and the network model and sends it into the *C-Analysis*.

The *C-Monitoring* function can use multiple AI techniques to achieve a zero-touch behavior [57]. In this via, we encourage the use of 1P2L for accomplishing intelligent probing. A starting point can be IPro, an RL-based intelligent probing solution presented by [58]. IPro allows changing the monitoring frequency (*space of actions*) autonomously in seeking for achieving, for instance, a trade-off between the monitoring bandwidth overhead (*space of states*) and the precision of measurements (*reward*). As stated by the authors, IPro has limitations related to its convergence time. In this sense, we consider that AP can be used to reduce that time and, as a result, optimizing IPro.

*C-Analysis* mainly performs three processes. First, it analyzes the management *policies* obtained from the *KB* and evaluates their accomplishment according to the network status obtained from *C-Monitoring* (e.g., if an SLA has been breached). Second, *C-Analysis* updates the network status in the *KB*. Finally, this function triggers the *C-Plan* to achieve one or a set of goals, such as *optimize the usage of the links* or *decrease the energy consumption*. Overall, achieving the goal involves coming up with possible solutions to detected problems. We consider that *C-Analysis* must also offer zero-touch network and service management capabilities [59] by using Big Data Analytics [60].

*C-Planning* groups AP and RL modules and can be combined diversely. For example, using the IXLP strategy as follows.

The model of the network (*domain*), the current *status* is retrieved from the *KB* and the *goal* from the *C-Analysis* module.

When no experience exists, *AP* computes a set of plans by simulating transitions using the network model (*domain*), the *goal*, and the *current status*. These plans are translated to experience (value of each state–action pair) in a *table* of *value function*. This experience (*value function*) is stored in the *knowledge* module for future use.

The *RL* agent uses this experience to decide the following actions to perform in order to achieve the *target state (goal)*. From this information, RL starts learning by interacting with the network (*environment*), and the actions are performed in the *C-Execute* function. This module also receives the *Reward* from the *C-Monitoring* module.

*C-Execute* carries out the actions defined by C-Plan. These actions can be low-level (e.g., change antenna azimuth and provision a particular Linux flavor) or high-level (e.g., provision a network slice for remote surgery). As high-level actions are not executable directly in the network, they must be decomposed up to obtain only low-level tasks using, for instance, HTN. The execution of these actions receives a *reward* that provides feedback to the entire loop’s functioning.

From a high-level perspective, the loop of the proposed architecture operates as follows. Information on the status of the managed network is collected by the *C-monitor*, which sends this information to the *C-Analysis* function. *C-Analysis* updates the information on the network status in the *KB*. In addition, the *C-Analysis* function defines a new *goal* and notifies the *C-Planning* function. Depending on the strategy selected (e.g., 1P2L), this function comes up with actions that are executed by *C-Execute* and may change the status of the *Managed Network*. This new status is monitored by the *C-Monitoring* function and reported to the *RL* module of *C-Planning* through the *C-Analysis* and the *KB* functions, thus closing the loop. This loop continues until the the goal state is achieved.

## 6. Case Study: Admission Control for Network Slicing Based on AP and RL

This case study’s aims are two-fold. First, it presents an architecture that follows the 1P2L strategy for performing network slicing admission control. Second, it evidences the impact on the rewards and convergence of using AP and RL jointly by comparing the 1P2L-based architecture prototype with solutions based on RL and DRL.

### 6.1. Network Slicing Admission Control with 1P2L

Network Slicing is pivotal for achieving 5G and beyond networks since it provides the capability of creating isolated logical networks known as Network Slices (NSL), each one with different QoS requirements. Admission Control (AC) is the process that allows Network Service Providers to verify if available physical and virtual resources in the network infrastructure are sufficient to respond to the NSL Requests (NSLR) coming from multiple tenants [61].

**Figure 3:**
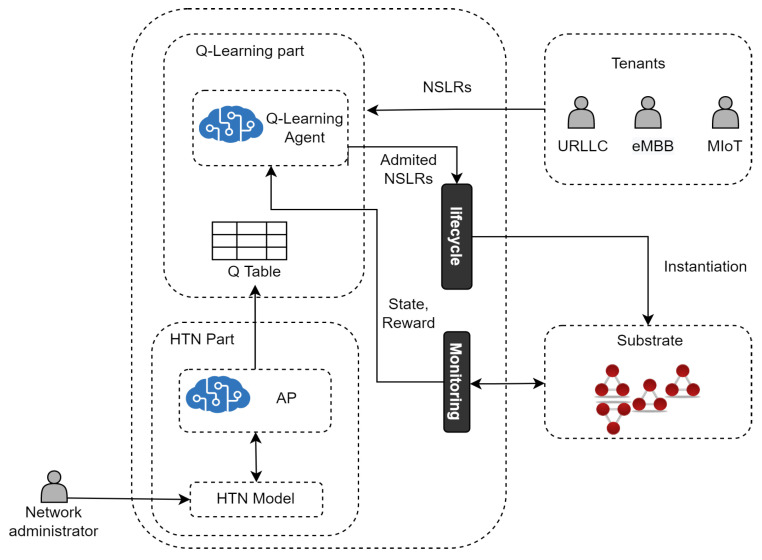
HTN Planning and Q-learning for Network Slicing Admission Control.

Figure 3 introduces an architecture based on 1P2L for Network Slicing Admission Control. In particular, this architecture realizes 1P2L using a Model-Based Reinforcement Learning (MBRL) approach instantiated by Q-learning and HTN. Specifically, Q-learning is used to learn optimal admission policies for NLSRs, and HTN is used to represent a model of the Network. HTN populates the Q-table to speed up the learning process of the Q-learning agent.

***The Q-learning part of the architecture*** learns the optimal NSLs admission policy by interacting with the environment and operates according to our previous work [62], called SARA, that provides a solution for admission control of 5G Core NSLs. Aiming at making this paper self-contained and providing the 1P2L architecture comprehension, in what follows, we include SARA’s descriptive information. From a high-level perspective, as in SARA, in the learning part of the 1P2L approach:

- The Q-learning agent first receives NSLRs and makes decisions (i.e., execute actions) on their admission.
- The Admitted NSLRs are instantiated in the substrate network by the Lifecycle Module.
- The Q-learning agent receives a reward and an updated state from the Monitoring Module. A state is defined by the tuple {cpu(E),cpu(C),bw(L)} (where cpu(E) and cpu(C) are the available processing capacity in the set of edge (*E*) and core nodes (*C*), respectively, and bw(L) is the available bandwidth in the set of links (*L*)) and represents the available resources in the substrate network after the Q-learning agent executes an action. Each action is represented by $a=\{pct_{embb},pct_{urllc},pct_{miot}\}$, where $pct_{embb}$, $pct_{urllc}$, and $pct_{miot}$ are the percentages to admit for each type of service.
- The Q-learning agent chooses to execute the action *a* that returns the maximum accumulated reward (i.e., the Q-value with the highest profit) while optimizing resource utilization. The reward is a profit function calculated by subtracting the amount of money earned from selling the NSL, minus the operational cost caused by using processing and bandwidth resources for running the NSL in the Substrate Network. The quality of the action is determined based on the maximization of monetary profit generated by taking that action.
- The Q-learning agent goes to the next state and stores each action’s Q-value (profit) in the Q-table.

***The HTN part of the architecture*** aims to speed up the Q-learning process by MBRL. The Dyna framework [14] is a commonly and often used solution for realizing MBRL. Dyna’s fundamental idea is to employ the experience to construct a model of the environment and then use this model for updating the value function (i.e., populate the Q-table) without having to interact with the environment [63].

**Figure 4:**
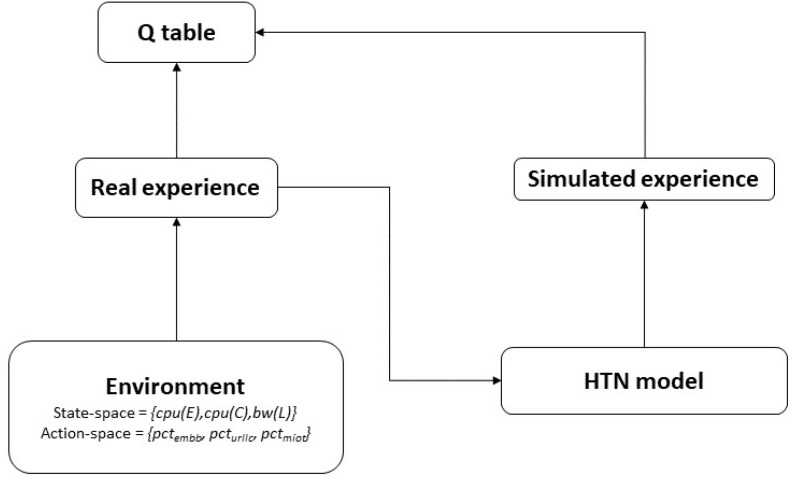
Dyna framework [63].

In particular, based on the experience obtained after running the learning part of the architecture in an isolated way (see Figure 4), we built a model of the environment described using HTN.

As mentioned above, this HTN model is used to populate the Q-able and speed up the learning process (see Figure 3).

Listing 1 depicts some parts of the model described in HTN. For example, the compound task *optimize_edge_20* requires that the available capacity is 20 and has some substasks associated with the capacity of edge, core nodes, and BW links. As can be seen, this listing is more understandable for humans than a Q-table or a Neural network. In this prototype, the Model was created by hand, but it is possible to automate this creation.

The environment model was built using HTN planning, which is similar to the Q-table structure of the RL agent.

Different scenarios were created and the HTN planner was used to simulate the interactions with the environment. This experience was mapped from HTN to the Q-Table and is used by the RL agent during its learning process.

To carry out the experiments with this architecture, we first developed the modules of the prototype of the 1P2L-based architecture, called from now on HTN-based Q-learning agent, with Python 3. Second, we built a discrete event simulator for network slicing admission control using Python3. Third, we deployed in such a simulator a 16-node network topology generated by using the Barabasi–Alberth algorithm [21]. This network was composed of 4 core nodes and 12 edge nodes with capacities of 300 and 100 processing units, respectively. All substrate links had a capacity of 100 bandwidth units. Fourth, we performed experiments on an Ubuntu 16.04 LTS desktop with an Intel Core i5-4570 CPU and 15.5 GB RAM. Fifth, using this setup, we measured the reward obtained by the HTN-based Q-learning agent prototype when the number of tasks in the model is 1, 2, and 4.

**Listing 1:** HTN Model for Network Slicing Admission Control.

task: optimize_edge_20 (edge,central,bw) precond: capacity (edge,20) subtasks: assign_capacity (edge,50), assign_capacity (central,100), assign_capacity (bw,50)

task: optimize_edge_40 (edge,central,bw) precond: capacity (edge,40) subtasks: assign_capacity (edge,75), assign_capacity (central,100), assign_capacity (bw,50)

task: optimize_central_20 (edge,central,bw) precond: capacity (edge,40) capacity (central,20) subtasks: assign_capacity (edge,75), assign_capacity (central,100), assign_capacity (bw,50)

task: optimize_edge_60 (edge,central,bw) precond: capacity (edge,40) subtasks: optimize_central_20 (edge, central, bw)

**Figure 5:**
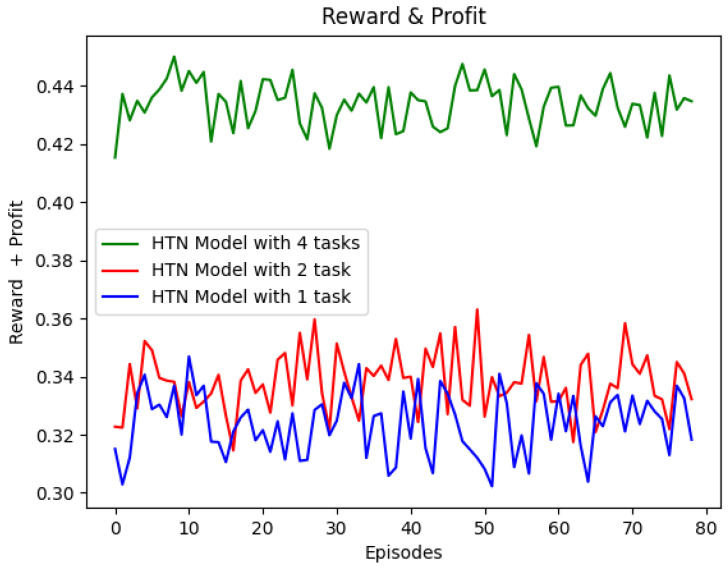
Results for different tasks.

Figure 5 shows that the reward obtained by the HTN-based Q-learning agent increases with the number of HTN tasks. The reward achieved using an HTN model with one task is 0.31 as a minimum and 0.34 as a maximum, whereas the reward obtained using two tasks in the model starts in 0.32 and reaches a maximum value of 0.36. When the agent uses the HTN model with four tasks, it reaches values of reward between 0.42 and 0.45. As expected, a more granular model allows the HTN-based Q-learning agent to obtain better results because the agent starts to learn with a Q-table initialized with more action-state values, and therefore the number of iterations needed to learn is lower.

#### 6.1.1. Convergence

To evaluate the convergence in the discrete event simulator, we deployed the 16-node network topology described above. Using this setup, we measured the minimum number of episodes that the Q-learning (SARA) and HTN-based Q-learning (i.e., the 1PRL prototype) agents need to obtain the maximum reward possible stably.

**Figure 6:**
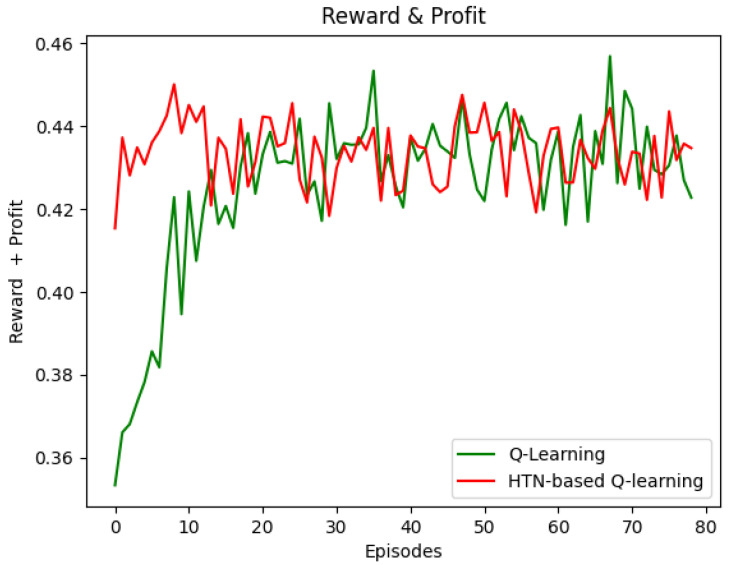
Convergence time—Reward vs. Episodes.

Figure 6 shows that the HTN-based Q-learning agent converged faster than the Q-learning agent did. The HTN-based Q-learning agent achieved the maximum reward in the seventh episode approximately (33 s). Conversely, the Q-learning agent obtained the maximum reward in about 35 episodes (185 s). These results are because the HTN model simulates the environment and defines values for the actions. Thus, when the HTN-based Q-learning agent starts, the learning time can be reduced substantially; it is noteworthy that the minimum reward obtained with the HTN-based Q-learning (0.42 approximately) at the beginning is the same as Q-learning obtained after the eighth episode (36 s). This reduction could significantly benefit processes needing real-time decision making, such as the AC process.

#### 6.1.2. 1P2L vs. Deep Reinforcement Learning

The 1P2L prototype is also compared to that given by DSARA [62] regarding reward and convergence. DSARA is a solution that uses Deep Q-Learning [26,64], Experience Memory Replay [65,66], and two Neural Networks (Target and Online) to perform efficient network slicing admission control.

**Figure 7:**
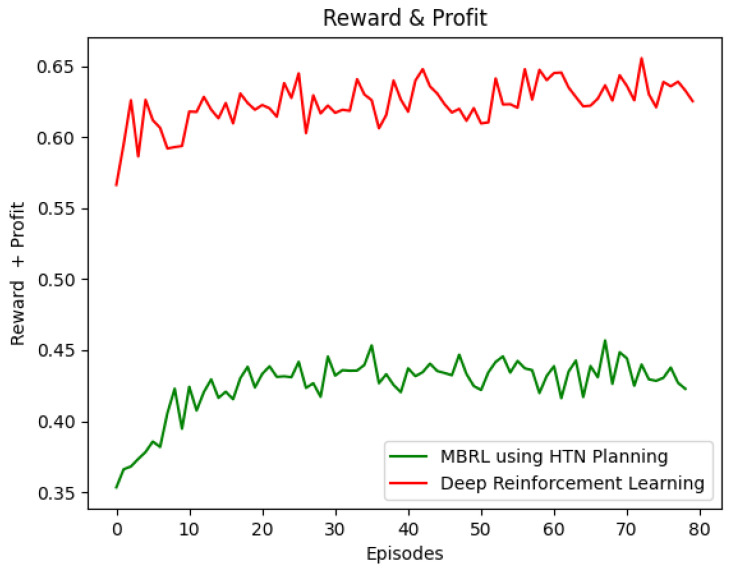
DeepRL vs. Model-based RL.

Figure 7 depicts the reward obtained using the DRL and HTN-based Q-learning agents. As expected, the former outperforms the latter regarding the reward obtained. The DRL approach can be used in scenarios where the number of states and actions is vast. Unlike DRL, which makes black-box decisions, the 1P2L approach starts to learn from an HTN-based white-box model that can be created using the experience of the Network Administrator. Consequently, the HTN-based Q-learning agent makes human-readable and straightforward decisions.

Limitations of this study includes the size of the problem, the availability of data and the knowledge required for HTN planning. First, this case-study models the few domain tasks; in real higher scenarios, the complexity of size of the domains may be unmanageable. Second, we modeled the HTN domain using the data from previous executions; however, several scenarios cannot be adapted to the hierarchical nature of HTNm and there could be scarce data or experience from the administrator. Third, although the modeling of the HTN can represent an alternative to complex NN models, this modeling requires a technical knowledge on automated planning.

## 7. Conclusions

This paper showed the use of AP for improving RL in the context of functional network management areas and proposed an architecture to realize a cognitive management control loop by combining AP and RL. We evaluated the proposed solution by using a prototype that combined AP and RL framed in MBRL using Q-learning and HTN. Evaluation results of the MBRL-based prototype in a simulated environment evidenced that the combination proposed improves RL but demonstrates lower performance than DRL regarding the reward and convergence time metrics. Consequently, we encourage the use of AP-based RL in scenarios with small datasets, where the expert’s knowledge can be represented in AP domains.

When compared with Q-Learning, our MBRL-based approach achieves very good results in only one episode, while Q-Learning achieves similar results in eight episodes. Each episode implies a set of trial-and-error iterations, which may be prohibitive in production scenarios. Conversely, the performance of neural networks is much higher than the MBRL-based approach; this is due to the fact that the recent algorithms can achieve models with high precision. However, these models cannot be understood internally, and the data and computational effort can make it difficult to apply this approach in several scenarios.

The main impact of our research is the reduction in the learning time of the reinforcement learning algorithms in the context of network management. This is achieved without the need of big datasets and high processing time. In addition, this method allows modeling the environment in HTN models that may be more human-understandable than complex neural networks. These benefits make RL closer to being used in real scenarios.

There are a number of compelling future directions to this work. First, we have explored the feasibility of combining AP with Q-Learning. Our future work will focus on using other RL approaches and exploring the integration with DRL [67]. Second, as AP requires significant human expertise before it can be applied to new problems and domains. Our future work will focus on exploring the automatic generation of AP problems and domains from natural language [30]. Third, we also plan to implement and evaluate our AP-based MBRL method in other network management scenarios such as traffic control and classification [68] and intrusion detection [69]. We will compare our results with datasets from these new scenarios. We leave these problems as future work.
